# Headache Characteristics in the Neurological Emergency Department: A Retrospective Study

**DOI:** 10.3389/fneur.2021.706074

**Published:** 2021-08-19

**Authors:** Florian Rimmele, Josephine Janke, Peter Kropp, Annette Grossmann, Till Hamann, Uwe Walter, Tim P. Jürgens

**Affiliations:** ^1^Department of Neurology, Headache Center North-East, University Medical Center Rostock, Rostock, Germany; ^2^Institute of Medical Psychology and Medical Sociology, University Medical Center Rostock, Rostock, Germany; ^3^Institute of Diagnostic and Interventional Radiology, Pediatric Radiology and Neuroradiology, University Medical Center Rostock, Rostock, Germany

**Keywords:** headache, emergency department, primary headache, migraine, secondary headache

## Abstract

**Background:** The care of patients with headache in the emergency department (ED) represents a diagnostic and clinical challenge. Data on the prevalence and characteristics of headache patients in purely neurological EDs are sparse. The aim of the present study is to examine patient profiles with the cardinal symptom of headache in an academic neurological ED, to analyze correlations between headache characteristics and search for differences compared to the interdisciplinary ED.

**Methods:** This retrospective cross-sectional study assessed all patients who presented to the ED of the Department of Neurology at Rostock University Medical Center between November 2013 and November 2016 with the main symptom of headache. Epidemiological, clinical, diagnostic data as well as key data regarding the care structure were recorded. Correlations between headache characteristics and diagnosis at discharge were analyzed and risk profiles were identified using binary logistic regression analysis.

**Conclusion:** This study comprehensively characterized a large collective of patients with the cardinal symptom of headache presenting to a purely neurology emergency department.

## Background

Headaches are a frequent complaint and represent a major challenge in the emergency department (ED). They result in 1–4 % of all presentations in an interdisciplinary ED (1–4) and are one of the most common presenting symptoms in emergency neurological patients, accounting for 8.4–16% of visits (5). According to Goldstein et al., 2.2% of patients in American interdisciplinary EDs presented with headaches based on data of the National Hospital Ambulatory Medical Care Survey (1). In a study by Doretti et al., headaches accounted for 3.2% of visits to two EDs in Italy and Austria (6). In the interdisciplinary ED of a German university hospital, headaches were found caused 3.5% of all visits and 19% of the primary neurological presentations (7). Besides headaches, the most frequent chief neurological complaints in an interdisciplinary ED are vertigo, epileptic seizures and motor deficits (8). The prevalence of headaches in the general population is high: 5–15% for migraine, 38% for tension-type headaches, and 4% for chronic headaches (9). Only 7% of patients with migraine seek headache-specific help at the emergency department (9). As headaches

can be secondary to a serious, potentially life-threatening disease, the major challenge in the ED is to identify the patients who require further diagnostics and rapid specialist treatment from among those with the purely primary headaches. If deemed primary, the correct diagnosis according to the ICHD-3 criteria (10) is essential for correct treatment for headache specialists and neurologists, while it may be more important not to miss a secondary course for emergency physicians. Several studies have shown that primary headaches are under- and misdiagnosed in the ED inferring inadequate treatment (11–13). The proportion of secondary headaches in the ED varies from 4% to over 14% for severe headache with sudden onset (1). Various guidelines support the ED physician in the choice of additional diagnostic procedures (14–16). The concept of warning symptoms (“red flags”) has proven helpful as a decision aid. Typical “red flags” in the clarification of headaches are patient age over 50 years, immunosuppression, an abnormal neurological examination finding or additional symptoms such as fever. However, a study by Goldstein et al. 2018 also showed that a normal neurological finding in patients with headaches does not rule out a secondary headache and pathological brain imaging findings (17). The care of patients with headache in the ED represents a challenging and cost-intensive task, especially in times of increasing demand by patients (18, 19). Data on patient's characteristics in purely neurological EDs are sparse. The aim of the present study is to examine patient profiles with the main symptom of headache in a neurological ED at an academic tertiary center, and to record clinical, epidemiological as well as diagnostic data. Key data regarding the care structure are presented, correlations between headache characteristics are examined, predictors of symptomatic headaches are determined, and risk profiles for individual headache groups are developed.

## Methods

This retrospective cross-sectional study assessed all patients aged at 18 years or above who presented to the neurological ED at Rostock University Medical Center between November 2013 and November 2016 with headache as main complaint. Data on administrative details were collected from the admission books for all patients. Then, the digital doctor's letters were screened for additional demographic information, previous medical history, headache characteristics, concomitant symptoms, instrumental diagnostic procedures, diagnosis at discharge. The diagnoses at discharge were categorized according to the ICHD-3 as primary and secondary headache (10). The data to be analyzed were transferred into an Excel table and a descriptive analysis of patient characteristics was conducted. The categorical data were summarized in numbers and percentages, and the continuous data according to means and standard deviations. The descriptive and exploratory statistical analyses were conducted using the statistical program SPSS 22 (IBM) and Excel (Microsoft). Differences in frequency distributions and the mean value of individual variables within comparison groups were examined using the Chi2 test or *t*-test. In case of ordinally scaled variables, the Kruskal-Wallis or Mann–Whitney *U* test were used. The statistical significance was defined as *p* <0.05.

Risk profiles were developed using binary logistic regression analysis. The final diagnoses primary and secondary headache served as dependent variables as well as the independent variables age, sex, medical history (cardiovascular, oncological, autoimmune/ rheumatological, trauma, etc.), specification of headache (duration of headache, pain intensity, new onset, pain character) and concomitant symptoms (such as nausea, fever, photo-, phonophobia etc.) as possible influencing factors. The models used were highly significant with *p* <0.001 and had a predictive value between 0.114 and 0.311 according to Nagelkerke (*R*^2^).

The research was conducted according to the Declaration of Helsinki. The retrospective, fully anonymized analysis of data was approved by the Ethics committee of the University Medical Center Rostock (identifier: A 2018/0168).

## Results

### Patient Demographics and Characteristics

11,210 patients who presented to the neurological ED of the University Medical Center Rostock between November 2013 and November 2016 were included in the study. Thousand and twenty patients suffered from headache as their cardinal symptom. Thus, the prevalence of patients with headache in the ED was 9.1%. Their average age was 46 years (SD 19.1; range from 18 to 98) and women were more frequently affected than men (64 vs. 36%). Of the 1,020 patients, 296 (29%) had a secondary headache. These patients had a mean age of 52 years (SD 19.7 range from 19 to 88) and were older than the overall cohort of patients with headaches; 60.1% were female and 39.1% were male. Some 412 (40.4%) patients had no previous medical history. Of the various pre-existing conditions, central nervous system disorders were the most common (33.2%, *n* = 339), of which 171 patients (16.8%) had a known history of migraine. The second most frequent pre-existing conditions were cardiovascular diseases (22.9%; *n* = 234), and the third most frequent were concomitant endocrine diseases (12.8%; *n* = 131). The majority of patients presented to the ED with a referral or admission slip from their general practitioner/ or ambulatory-care specialist physician (51.5%, *n* = 525), followed by self-presentation (20.2%; *n* = 206) and presentations through emergency medical services (21%, *n* = 214). There was a significant seasonal peak of presentations to the ED in June (*p* = 0.005) and diurnal peak between 10 am and 1 pm (*p* < 0.001). The average length of stay of patients in the ED was 4.99 h (SD 3.8; median 4 h) and 9.6% (*n* = 98) of patients were admitted to ward for further inpatient treatment. The demographic data and medical history of patients with the cardinal symptom of headache in the ED are summarized in Table 1.

**Table 1:** Demographics and characteristics of patients with headache as cardinal symptom in the ED.

	**Headache *n* = 1,020 (9.1%)**	**Primary headache *n* = 409 (40.1%)**	**Secondary headache *n* = 296 (29%)**
*Demographics*			
Age (y) [SD]	46 [19.1]	40.8 [16.4]	51.6 [19.7]
Age, range (y)	18–98	18–91	19–98
Female, *n* (%)	653 (64)	281 (69)	178 (60)
Male, *n* (%)	367 (36)	128 (31)	118 (40)
*Medical history*			
Negative, *n* (%)	412 (40.4)	170 (41.2)	111 (37.5)
CNS-Disease, *n* (%)	339 (33.2)	174 (42.4)	72 (24.3)
known Migraine, *n* (%)	171 (16.8)	132 (32.3)	20 (6.8)
Cardiovascular Disease, *n* (%)	234 (22.9)	55 (13.4)	91 (30.7)
Endocrine Disease, *n* (%)	131 (12.8)	41 (10)	42 (14.2)
Head trauma, *n* (%)	37 (3.6)	1 (0.2)	22 (7.4)
Oncological Disease, *n* (%)	45 (4.4)	10 (2.4)	21 (7.1)
Respiratory Disease, *n* (%)	45 (4.4)	8 (2)	28 (9.5)
*Time of presentation*			
06:00–11:59, *n* (%)	321 (31.5)	130 (31.8)	90 (30.4)
12:00–17:59, *n* (%)	441 (43.2)	175 (42.8)	134 (45.3)
18:00–23.59, *n* (%)	199 (19.5)	82 (20)	56 (18.9)
00:00–07:59, *n* (%)	44 (4.3)	19 (4.6)	14 (4.7)
Data missing, *n* (%)	15 (1.5)	3 (0.7)	2 (0.7)
*ED length of stay*, h (mean) [SD]	4.99 [3.8]	4.44 h [ 3.6]	5.41 [ 4.3]
*Inpatient admissions* (*n* = 1,010), *n* (%)	98 (9.6)	6 (1.5)	64 (21.7)
*Referral to ED by*			
Ambulance/Emergency doctor, *n* (%)	214 (21)	87 (21.8)	79 (27.3)
General practitioner/Specialist, *n* (%)	525 (51.5)	196 (49.1)	151 (52.2)
Own initiative, *n* (%)	206 (20.2)	101 (25.3)	49 (17)
Other, *n* (%)	44 (4.3)	15 (3.7)	10 (2.3)
Missing	31 (3)	6 (1.5)	7 (2.4)

### Headache Characteristics

The most frequent headache characteristic was dull (25.8%, *n* = 263), followed by stabbing (7.9%, *n* = 81) and others (1.9%, *n* = 19), although in most cases, the character of the headache was not assessed/documented (57.9%, *n* = 591). The average pain intensity on the NRS (numerical rating scale) from 0 to 10 was 6.6 (SD 2.28). Stabbing headaches were significantly more frequent in the “Neuropathies and Facial pain” group (*p* < 0.001; for further details see Table 2). With regard to pain intensity, there was no statistically significant difference between the individual headache groups (*p* = 0.1). While a positive history of headaches was found in 34.2% (*n* = 349) patients, there was no history of headaches in 15.6% (*n* = 159) and no assessment respectively documentation in 50.2% (*n* = 512). In 78.9% (*n* = 805) of patients, concomitant symptoms such as nausea (33.6%, *n* = 343), light-headedness/vertigo (17.5%, *n* = 179), photo-/phonophobia (16.5%, *n* = 168) and visual disturbances (15.1%, *n* = 154) were recorded (see Table 2). The concomitant symptoms nausea, photo- and phonophobia and visual disturbances were significantly more frequent in the group with primary headache (*p* < 0.001).

**Table 2:** Headache description and accompanying symptoms.

	**All *n* = 1,020 (%)**	**Primary Headache *n* = 409**	**Secondary headache *n* = 296**	**Neuropathies and facial pain *n* = 32**	**Other headache disorders *n* = 31**	**No diagnosis given *n* = 252**
*Pain character*						
Dull	263 (25.8)	113(27.6)	75 (25.3)	5 (15.6)	8 (25.8)	62 (24.6)
Stabbing	81 (7.9)	41 (10)	13 (4.4)	5 (15.6)	1 (3.2)	21 (8.3)
Throbbing	66 (6.5)	46 (11.2)	12 (4.1)	1 (3.1)	4 (12.9)	3 (1.2)
other	19 (1.9)	10 (2.4)	3 (1)	2 (6.3)	1 (3.2)	2 (0.8)
missing	591 (57.9)	199 (48.7)	193 (66.2)	19 (59.4)	17 (54.8)	163 (64.5)
*Pain intensity* [SD]	6.64 [2.28]	6.91 [2.2]	6.18 [2.54]	6.86 [2.67]	7 [2.59]	6.49 [2.07]
missing	579 (56.8)	194 (47.4)	194 (65.6)	25 (78.1)	22 (71)	137 (56.4)
*Nausea*	343 (33.6)	187 (45.7)	80 (27)	2 (6.3)	12 (38.7)	62 (24.6)
*Dizziness*	179 (17.5)	64 (15.6)	54 (18.2)	1 (3.1)	8 (25.8)	52 (21.4)
*Photo-, Phonophobia*	168 (16.5)	131 (32)	20 (6.8)	2 (6.3)	3 (9.7)	12 (4.9)
*Visual disturbance*	154 (15.1)	103 (25.2)	26 (8.8)	4 (12.5)	2 (6.5)	19 (7.8)
*Fever*	46 (4.5)	5 (1.2)	26 (8.8)	-	-	15 (6)
*Valsalva, Sneezing, Coughing*	32 (3.1)	6 (1.5)	19 (6.4)	-	1 (3.2)	6 (2.4)
*Known Headache*						
yes	349 (34.2)	223 (54.5)	61 (20.6)	6 (18.8)	8 (25.8)	51 (20.2)
no	159 (15.6)	80 (19.6)	30 (10.1)	3 (9.4)	4 (12.9)	42 (17.3)
missing	512 (50.2)	106 (25.9)	205 (69.3)	23 (71.8)	19 (61.3)	159 (63.1)
*Headache onset*						
minutes	1 (0.1)	-	-	-	-	1 (0.4)
hours	368 (36.1)	179 (43.8)	94 (31.8)	8 (25)	12 (38.7)	75 (29.8)
days	360 (35.1)	130 (31.8)	116 (39.2)	17 (53.1)	8 (25.8)	89 (35.3)
weeks	107 (10.5)	33 (8.7)	39 (13.2)	2 (6.3)	3 (9.7)	30 (12.3)
month	47 (4.6)	19 (4.6)	11(3.7)	3 (9.4)	1 (3.2)	13 (5.3)
years	35 (3.4)	18 (4.4)	7 (2.4)	-	1 (3.2)	9 (3.7)
missing	102 (10)	30 (7.3)	29 (9.8)	2 (6.3)	6 (19.4)	35 (13.9)
*Duration of headache (attacks)*						
seconds	20 (2)	5 (1.2)	3 (1)	4 (12.5)	-	8 (3.3)
minutes	58 (5.7)	26 (6.4)	8(2.7)	5 (15.6)	-	19 (7.5)
hours	335 (32.8)	186 (45.5)	68 (23)	4 (12.5)	11 (35.5)	66 (26.2)
days	227 (22.3)	71 (17.4)	79 (26.7)	10 (31.3)	7 (22.6)	60 (23.8)
weeks	48 (4.7)	12 (2.9)	18 (6.1)	1 (3.1)	-	17 (7)
months	9 (0.9)	1 (0.2)	4 (1.4)	-	-	4 (1.6)
years	1(0.1)	-	1 (0.3)	-	-	-
missing	322 (31.6)	108 (26.4)	115 (38.9)	8 (25)	13 (42)	78 (31)

### Additional Diagnostics

Cerebral imaging was performed in 69% (*n* = 704) of patients: 39 % (*n* = 398) received cerebral magnetic resonance imaging (head MRI) and 28.8 % (*n* = 294) received cerebral computed tomography (head CT). The head MRI yielded abnormal findings in 9.3 % (*n* = 37) of cases and the head CT yielded abnormal findings in 9.5 % (*n* = 28) of cases. In 12.9% (*n* = 132) of patients, cerebrospinal fluid (CSF) was analyzed, which was abnormal in 2.3% (*n* = 3) of cases. Duplex sonography of the cervical and cerebral arteries amounted to 8.3% (*n* = 82), with abnormal findings in six patients (7.3%), followed by electroencephalography (EEG) in 5% (*n* = 51) of patients with abnormal results in 8 patients (15.7%). For a summary of additional diagnostics see Table 3.

**Table 3:** Additional diagnostics.

**Investigations**	**Headache in the ED *n* = 1,020 (%)**	**Abnormal findings *n* = 83 (%)**
None		
Cerebral MRI, *n* (%)	*398 (39.0)*	37 (9.3)
Cerebral CT, *n* (%)	294 (28.8)	28 (9.5)
Cerebrospinal fluid (CSF) analysis, *n* (%)	132 (12.9)	3 (2.3)
Duplex sonography of extra- and intracranial arteries, *n* (%)	82 (8.3)	6 (7.3)
Electroencephalography (EEG), *n* (%)	51 (5.0)	8 (15.7)
Other	4 (0.4)	1 (0.1)

### Headache Diagnoses

The final diagnoses according to the ICHD-3 classification are summarized in Table 4. Primary headaches were the most prevalent headache type (40.1%, n = 409), with migraine as the most frequent subtype (73.4%), followed by tension-type headache (17.2%) and trigeminal autonomic cephalalgias (7.7%). Trigeminal neuralgia was the most common type of facial pain, at 3.1% (*n* = 32). Patients with secondary headache formed the second-largest group, with 29% (*n* = 296) of patients. In 31 (3.0%) of patients, other headache disorders were diagnosed, and a total of 252 (24.7%) of patients received no final diagnosis. Of the secondary headaches, 17.2% were attributed to infection, 17.2% to disorders of electrolyte dyshomeostasis, 16.2% to vascular disorders, 15.2% to non-vascular disorders, 5.7% to trauma, 7.4% to intoxication or substance withdrawal, and 11.1% to psychiatric disorders. Among the headaches attributed to vascular diseases, 20.8% (*n* = 10) emerged in patients with acute cerebral ischemia, 12.5% (*n* = 6) in patients with subarachnoid hemorrhage, 14.6% (*n* = 7) in patients with subdural hematoma and 6.3% (*n* = 3) in patients with arterial vessel dissection.

**Table 4:** Final diagnosis at discharge.

**Type of headache (ICHD-3)**	**Headache in the ED *n* = 1,020 (%)**
*Primary headache*	*409 (40.1)*
Migraine, *n* (%)	303 (73.4)
Tension type headache (TTH), *n* (%)	71 (17.2)
Trigeminal autonomic cephalalgias (TAC), *n* (%)	32 (7.7)
Other primary headache	3 (1.7)
*Secondary headache*	296 (29)
Headache attributed to trauma, *n* (%)	17 (5.7)
Headache attributed to vascular disorders, *n* (%)	48 (16.2)
Cerebral Ischemia, *n* (%)	10 (20.8)
Subarachnoid hemorrhage, *n* (%)	6 (12.5)
Subdural hematoma, *n* (%)	7 (14.6)
Aneurysm/dissection of brain supplying arteries, *n* (%)	3 (6.3)
Transient ischemic attack (TIA), *n* (%)	5 (10.4)
Cerebral sinovenous thrombosis, *n* (%)	1 (2.1)
Stroke unspecified, *n* (%)	10 (20.8)
Giant cell arteritis, *n* (%)	3 (6.3)
Headache attributed to non-vascular disorders, *n* (%)	45 (15.2)
Headache attributed to substances or withdrawal, *n* (%)	22 (7.4)
Headache attributed to infection, *n* (%)	51 (17.2)
Headache attributed to disorders of homeostasis, *n* (%)	51 (17.2)
Headache attributed to disorders of facial or cervical structure, *n* (%)	29 (9.8)
Headache attributed to psychiatric disorders. *n* (%)	33 (11.1)
*Neuropathies and Facial pain, n* (%)	*32 (3.1)*
*Other headache disorders, n* (%)	*31 (3.0)*
*No diagnosis given, n* (%)	*252 (24.7)*

### Advanced Analyses

A comparison of age showed that patients with a primary headache were significantly younger than patients with a secondary headache (*p* < 0.001). Gender was unrelated to secondary headache, but in the group of primary headaches, migraines were more frequent in women (*p* < 0.001) while trigeminal autonomic headaches were more frequent in men (*p* < 0.001). With regard to the characteristics of headaches, which were only assessed in a total of 48% of the patients, it was apparent that stabbing headaches occurred significantly more frequently in the group of neuropathies and facial pain (*p* < 0.001). The concomitant symptoms of nausea, photo-, phonophobia and visual disturbances (*p* < 0.001) occurred more often in the group of primary headaches, while no significant difference was found between the groups regarding pain intensity. In patients with cardiovascular diseases, secondary headache were more prevalent (*p* < 0.0001). Patients with primary headache had a higher risk of having a history of CNS diseases (*p* < 0.0001). Of note, known migraine was also subsumed thereunder. A positive history of cerebral infarction was associated with a missing final headache diagnosis (*p* = 0.017). There was a significant peak of seasonal presentations to the ED in June (*p* = 0.005; see also Table 5) as well as diurnal presentations between 10 am and 1 pm (*p* < 0.001). Patients who self-presented to the ED were more likely to suffer from trigeminal neuralgia and facial pain (*p* = 0.001). The diagnosis of secondary headache was associated with inpatient admission of the patient (*p* < 0.001).

**Table 5:** Average number of patient-presentation per month.

**Month**	**Total number**	**Headache patients (percent)**	**Monthly proportion of headache patients**
January	919	85 (9%)	8.3%
February	796	80 (10%)	7.8%
March	871	82 (9%)	8%
April	827	99 (12%)	9.7%
May	914	86 (9%)	8.4%
June	907	115 (13%)	11.3%
July	927	97 (10%)	9.5%
August	874	89 (10%)	8.7%
September	870	64 (7%)	6.3%
October	900	73 (8%)	7.2%
November	853	66 (8%)	6.5%
December	884	81 (9%)	7.9%
Missing	668	3	

For the subsequent analysis of possible risk factors for secondary headache vs. primary headache, only patients with a formal headache diagnosis were considered. The risk of suffering from a secondary headache increased by 1.03 with every year of age (*p* < 0.001). A person aged 50 years or above is 2.85 times more likely to suffer from a secondary headache than someone aged below 50 years (*p* < 0.001), see Table 6.

**Table 6:** Analysis of possible risk factors for secondary headache versus primary headache.

**Model 1 Concomitant symptoms (highly significant, Nagelkerke 0.31)**					
**Concomitant symptoms**	**Regression coefficient B**	**Significance p**	**Exp (B)**	**95% confidence interval for EXP (B)**	
				**Lower value**	**Upper value**
Nausea	−0.477	0.011	0.621	0.429	0.898
Fever	2.295	<0.001	9.927	3.389	29.083
Valsava/Cough/Sneeze	2.023	< .011	7.565	2.462	23.244
Autonomic accompanying symptoms	−21.324	0.998	0.000	0.000	
Photo-, Phonophobia	−1.971	<0.001	0.139	0.079	0.244
Visual disturbance	−1.381	<0.001	0.251	0.152	0.415
**Model 2 Medical history (highly significant, Nagelkerke 0.199)**					
	**Regression coefficient B**	**Significance p**	**Exp (B)**	**95% Confidence interval for EXP (B)**	
**Disease in the medical history of**				**Lower value**	**Upper value**
Respiratory system	1.279	0.003	3.592	1.547	8.337
Cardiovascular system	1.105	<0.001	3.019	2.004	4.548
Central nervous system	−0.954	<0.001	0.385	0.269	0.552
Trauma	3.722	<0.001	41.335	5.438	314.163
Oncological disease	0.978	0.018	2.660	1.181	5.990
**Model 3 Age and Sex (highly significant, Nagelkerke 0.114)**					
	**Regression coefficient B**	**Significance p**	**Exp (B)**	**95% Confidence interval for EXP (B)**	
				**Lower value Wert**	**Upper value**
Sex (m)	0.332	0.047	1.394	1.005	1.933
Age	0.032	< .001	1.033	1.024	1.042

Diseases of the respiratory system, cardiovascular system, oncological diseases or trauma in the medical history significantly increased the risk of secondary headaches, while a history of migraine significantly decreased it. Diseases of the respiratory system were associated with a 3.6-fold risk of secondary headache (*p* = 0.003), diseases of the cardiovascular system with a 3.0-fold (*p* < 0.001), oncological diseases with a 2.7-fold (*p* = 0.018) and trauma in the medical history with a 41.4-fold (*p* < 0.001) risk of secondary headache. For more information on the discharge diagnoses of patients with trauma in their medical history, see Supplementary Material 1. For patients with a previous neurological condition (migraine included), the risk of secondary headache was reduced by 0.39-fold (*p* < 0.001). Concomitant symptoms such as fever (9.93-fold, *p* < 0.001), aggravation of headache by sneezing/coughing or Valsalva maneuver (7.57-fold, *p* < 0.001) increased the risk of secondary headache, while symptoms such as nausea, photo-, phonophobia and visual disturbances were associated with a significantly lower risk (*p* = 0.01, *p* < 0.001, *p* < 0.001) of secondary headache, see Table 6. No significant association was found between secondary headache and data on pain character, pain intensity, suddenness of onset, duration of headache or previously experienced headache. Also, no association was found with the type of referral and presentation.

## Discussion

This retrospective cross-sectional study comprehensively examined a large sample of patients presenting to a purely neurological emergency department and included 11,210 patients who presented between November 2013 and November 2016. A prevalence of 9.1% for headache as the cardinal symptom with a preponderance of female patients and an average age of 46 years was found. The prevalence reported in the literature ranged from 2.2% for North American EDs, 3.2% for hospitals in Austria and Italy, to 3% in a study from the UK (1, 6, 20). However, these frequencies pertained to interdisciplinary EDs and not a purely neurological ED as in the present study. An analysis of patient cohorts presenting to an interdisciplinary ED of a German university medical center found a headache prevalence of 3.5%, although the prevalence was substantially higher (19%) when only neurological cases were considered (7). Primary headaches were the most frequent headache type found in our study. Of these, migraine was the most frequent subtype, followed by tension-type headache and trigeminal autonomic cephalalgias. Accordingly, of all patients with headache in the ED, 29.7% suffered from migraine, which is in line with the rates of 26% (5) or 23% (21) described in other studies. A large North American epidemiological study which evaluated the data of the National Hospital Ambulatory Medical Care Survey found a higher rate of migraine patients in the ED, at 63% (1). This is probably due to the specific characteristics of the North American health system with its restricted access to outpatient care, and its reimbursement (6). The proportion of patients with secondary headache was remarkably high in our study (29%) compared to the very low secondary headache rates of 2% in interdisciplinary EDs described in the literature (1). Furthermore, a low ratio of primary to secondary headache of 9:1 was reported (7). In general, the rate of secondary headache in the ED is assumed to range between 5 and 22% (5, 22, 23). However, the reported rates are only comparable to a limited degree due to differences in the examined patient samples, corresponding health care systems, referral rates to the ED, and the differing definitions of secondary headaches in the respective studies. Nevertheless, an additional factor for the high proportion of secondary headaches in this study may be that this study was conducted in a purely neurological ED with a highly selected group of patients. Besides cerebral hemorrhage [six patients with subarachnoid hemorrhage; seven patients with subdural hematoma) there were also a high proportion of patients with cerebral ischemia (10 patients, and 10 patients with unspecified stroke as well as five patients with transient ischemic attacks (TIA)] in this group. The patients in this study with a discharge diagnosis of stroke had no obvious neurological deficits. The presenting symptom was headache. The diagnosis of stroke was made based on cerebral imaging, predominantly MRI imaging. It is a well-known finding that cerebral ischemia can be accompanied by headaches or can even be the leading clinical sign thereof. On the one hand, migraine with aura is associated with an approximately twofold increased risk of cerebral ischemia (24, 25) and on the other hand, a new-onset or concomitant secondary headache mimicking primary headaches can occur with cerebral ischemia, which is not yet sufficiently understood. The literature reports a wide range of 6–44% for the prevalence of headache associated with cerebral ischemia (26, 27). A meta-analysis by Harriott et al. found a pooled prevalence of 14%, with an increased risk of headache in the case of cerebral ischemia in the posterior circulation and for the female sex (27). An analysis of an Israeli stroke register over 2 months including 2,166 patients revealed that 8.4% had suffered from headache and cerebral ischemia, and a large proportion of 28% of patients suffered from TIA and headache (28, 29).

Detailed phenotypic data were documented in only 42% of patients, which is surprisingly low given that this information is essential for classifying the headache according to the current headache classification. This might also explain why no final diagnosis was reported in a large proportion (24.7%) of headache patients. Nevertheless, this value is still low compared to other assessments of headache patients in EDs, which reported rates of 32% (6), 36% (30) or even 44% (21) of cases in which no specific headache diagnosis was given. Primary headaches are often underdiagnosed or misdiagnosed in the ED (31). The presence/absence of important “red flags” such as thunderclap headache was also not systematically documented. Although it can be assumed that it was asked, the lack of documentation shows that standardized, structured documentation would be useful for headache patients in the ED. With regard to additional diagnostics, 69% of the patients in our study received cerebral imaging, of which 39% was made up of head MRI and 29% head CT. The high percentage of an emergency head MRI—in comparable studies 57.8% of patients received cerebral imaging but only 1.7% received head MRI (6)—is due to the high availability of MR imaging 24/7 at our department. Of the patients who underwent cerebral imaging, 9.4% had an abnormal finding, which corresponds to 6.4% of all patients with the cardinal symptom of headache. A study by Negro et al. assessing headache patients in an interdisciplinary ED in the period from 2015 to 2018 even showed that cerebral imaging was performed in 75% of cases, 6.6% of which yielded abnormal findings (32). By contrast, a large North American study which evaluated data from the National Hospital Ambulatory Medical Care Survey (NHAMCS) found that only 14% of patients with headache who visited an ED between 1992 and 2001 underwent cerebral imaging, which yielded a pathological diagnosis in 5.5% of cases (1). In our study, after head MRI and head CT, cerebrospinal fluid diagnostics were the third most frequent diagnostics, at 12.9% (*n* = 132), which yielded abnormal findings in three patients (2.3%). The literature describes even lower rates of cerebrospinal fluid diagnostics in patients with headache in the ED, of 1.2% (6), and the diagnostics should be reserved for a corresponding selected group of patients. The fourth most frequent diagnostic procedure was duplex sonography of the brain-supplying arteries, which 8.3% of the patients received and which led to an abnormal finding in 7.3% of them. This examination is also useful for selected patients, e.g., to detect an arterial dissection, which was found in our study in three patients (6.3% of secondary headaches), or also in the case of a suspected giant cell arteritis, which was likewise found in three patients (6.3% of secondary headaches). Guidelines and a concept of warning symptom “red flags” are classically used to help ED physicians with the challenging task of distinguishing between primary and secondary headache. (14–16) Munoz-Ceron additionally suggests predictors of a primary headache as “green flags” (33) based on the ICHD-3 classification.

In our study we also investigated which characteristics correlate with the diagnosis of primary or secondary headache. Binary logistic regression analysis showed that the risk of suffering from a secondary headache for a patient with headache in the emergency department increases 1.03-fold (*p* < 0.001) with each year of age. Similar findings were obtained by Locket et al., who described a higher risk of secondary headache in patients aged over 50 years (34), as well as Munoz-Ceron et al., who found an association of secondary headaches and immunosuppression in the medical history and age over 50 years with non-primary headache (33). We also found that a history of cardiovascular diseases, disease of the respiratory system, oncological diseases or trauma in the medical history were strongly correlated with the diagnosis of secondary headache. Trauma in the medical history was even associated with a 41-fold increased risk of secondary headache. Therefore trauma, cardiovascular and respiratory diseases in patient history should also be considered “red flags,” especially since they are not yet mentioned in corresponding guidelines, except for malignancy in the medical history (14–16). Munoz-Ceron et al. assume in their study that it is not meaningful to determine frequently observed symptoms in the case of primary headache in outpatient departments, such as nausea, vertigo, photo- and phonophobia and pain intensity, and found no statistically significant correlations with concomitant symptoms (33). However, in our sample the presence of nausea, photo- and phonophobia and visual disturbances significantly correlated with primary headaches (*p* = 0.011 and *p* < 0.001). In contrast, concomitant symptoms such as fever (9.93-fold, *p* < 0.001), worsening of headache by sneezing/coughing or Valsalva maneuvers (7.57-fold, *p* < 0.001) increased the risk of secondary headache. Another important point is that, contrary to common belief, headache intensity does not differ significantly between patients with primary and secondary headaches and is not a discriminatory criterion.

## Limitations

The study has several potential limitations that should be addressed. It was a retrospective cross-sectional data analysis with all problems inherent to its retrospective nature. Clinical data was collected in the ED in a busy clinical environment and documented without a uniform or standardized template in the patient record from where it extracted retrospectively. This procedure carries the risk of several systematic biases. A selection bias is, however, unlikely since the individual data records have been collected by many different doctors over a long period of time. Furthermore, this single-center study was conducted in a neurological ED in a university hospital of tertiary care and our results may not be generalized to other countries or to other EDs. Finally, a high proportion of patients presented to our neurological ED on their own initiative representing another source of bias. However, the rate of self-presentations was higher in patients with a final diagnosis of primary headaches than those with secondary headaches indicating that the patients‘ self-allocation was often purposeful.

## Conclusion

This study comprehensively characterized a large sample of patients with the cardinal symptom of headache presenting to a purely neurological emergency department. The majority of patients attending the ED because of headache were suffering from primary headaches. The unusually high proportion of secondary headaches (29%) may be due to the purely neurological setting with a high prevalence of cerebrovascular disorders. Higher age and a history of cardiovascular disease, respiratory disease, oncologic disease, or head trauma, as well as distinct concomitant symptoms such as fever, worsening of headache by sneezing/coughing or Valsalva maneuvers correlated positively with the diagnosis of secondary headache.

## Data Availability Statement

The original contributions presented in the study are included in the article/Supplementary Material, further inquiries can be directed to the corresponding author.

## Ethics Statement

The studies involving human participants were reviewed and approved by Ethikkommission an der Medizinischen Fakultät der Universität Rostock St.-Georg-Str. 108 18055 Rostock. Written informed consent for participation was not required for this study in accordance with the national legislation and the institutional requirements.

## Author Contributions

FR: drafted the manuscript, analysis, and interpretation and revising the manuscript. JJ: acquisition, analysis, interpretation, and revising the manuscript. PK: analysis, interpretation, and revising the manuscript. TH, AG, and UW: interpretation, and revising the manuscript. TJ: conception, analysis, interpretation and revising the manuscript. All authors contributed to the article and approved the submitted version.

## Conflict of Interest

FR served on advisory boards and/or as speaker for Allergan, Novartis, Teva, Ipsen. He has received royalties from Elsevier. PK served on advisory boards and/or as speaker for Allergan, Novartis, Teva and Lilly. UW has received speaker honoraria and travel funds from Ipsen Pharma, Merz Pharma, Allergan, Bristol-Myers Squibb, Daiichi Sankyo, Bayer Vital, Boehringer Ingelheim and Pfizer, and a research grant from Merz Pharma. He has received royalties from Thieme and Elsevier Press. He serves as editor of the European Journal of Ultrasound. TJ served on advisory boards and/or as speaker for Allergan, Autonomic Technologies, Desitin, Hormosan, Novartis, Lilly, Sanofi and Teva. The remaining authors declare that the research was conducted in the absence of any commercial or financial relationships that could be construed as a potential conflict of interest.

## Publisher's Note

All claims expressed in this article are solely those of the authors and do not necessarily represent those of their affiliated organizations, or those of the publisher, the editors and the reviewers. Any product that may be evaluated in this article, or claim that may be made by its manufacturer, is not guaranteed or endorsed by the publisher.

## Abbreviations

ED: Emergency department
ICHD-3: The International Classification of Headache Disorders 3rd edition
MRI: Magnetic resonance imaging
CT: Computed tomography
CSF: Cerebrospinal fluid
EEG: Electroencephalography
TIA: Transient ischemic attack
NHAMCS: National Hospital Ambulatory Medical Care Survey
ACEP: Guidelines on Acute Non-traumatic Headache Diagnosis and Management in the Emergency Department
CNS: Central nervous system
SD: Standard deviation
TTH: Tension type headache
TAC: Trigeminal autonomic cephalalgia.
