# Supplementary material for: Headache Characteristics in the Neurological Emergency Department: A Retrospective Study
Source: Front Neurol. 2021 Aug 19;12:706074. doi: 10.3389/fneur.2021.706074 (PMC8416997; doi:10.3389/fneur.2021.706074)
Supplement: Supplementary file 1 [file Table_1.docx]

**Supplement 1:** Final diagnosis at discharge for patients with head trauma in the medical history:

| Diagnosis at discharge | n | Percent |
| --- | --- | --- |
| Primary headache | 1 | 2.7 |
| Secondary headache | 22 | 59.5 |
| Neuropathies and Facial pain | 1 | 2.7 |
| Other headache disorders | 1 | 2.7 |
| No diagnosis given | 12 | 32.4 |

| Secondary headache | n | Percent |
| --- | --- | --- |
| Headache attributed to trauma or injury to the head and/or neck | 16 | 43.2 |
| Vascular disorder | 3 | 8.1 |
| Infection | 1 | 2.7 |
| Disorder of homoestasis | 2 | 5.4 |
